# Automated spectrometer alignment via machine learning

**DOI:** 10.1107/S1600577524003850

**Published:** 2024-06-20

**Authors:** Peter Feuer-Forson, Gregor Hartmann, Rolf Mitzner, Peter Baumgärtel, Christian Weniger, Marcus Agåker, David Meier, Phillipe Wernet, Jens Viefhaus

**Affiliations:** a Helmholtz-Zentrum Berlin für Materialien und Energie GmbH Albert-Einstein-Strasse 15 12489Berlin Germany; b Uppsala Universitet 751 05Uppsala Sweden; c MAX IV Laboratory Lund University PO Box 118 SE-22100Lund Sweden; European XFEL, Germany

**Keywords:** machine learning, X-ray diffraction, instrumentation, reflection zone plate

## Abstract

During beam time at a research facility, alignment and optimization of instrumentation, such as spectrometers, is a time-intensive task and often needs to be performed multiple times throughout the operation of an experiment. Despite the motorization of individual components, automated alignment solutions are not always available. In this study, a novel approach that combines optimisers with neural network surrogate models to significantly reduce the alignment overhead for a mobile soft X-ray spectrometer is proposed. Neural networks were trained exclusively using simulated ray-tracing data, and the disparity between experiment and simulation was obtained through parameter optimization. Real-time validation of this process was performed using experimental data collected at the beamline. The results demonstrate the ability to reduce alignment time from one hour to approximately five minutes. This method can also be generalized beyond spectrometers, for example, towards the alignment of optical elements at beamlines, making it applicable to a broad spectrum of research facilities.

## Abstract 2

The application of a machine learning approach for the automated alignment of a soft X-ray spectrometer is showcased. Our method has been validated with experimental data at the beamline and significantly reduces the alignment time from approximately one hour to just a few minutes.

## 1. Introduction

As part of our ongoing Röntgen–Ångström Cluster (RÅC) project aimed at developing a new soft X-ray spectrometer for fluorescence-detected absorption spectroscopy, we are conducting research to explore the application of machine learning methods in the design, development and automation of experiments at the BESSY II X-ray facility. The objective of our project is to conduct fluorescence-detected (partial-fluorescence yield) X-ray absorption spectroscopy at the 3*d* transition-metal *L*-edges, which are relevant for many important applications such as catalysis and many more applications (Chanda *et al.*, 2018 ▸; Aly *et al.*, 2016 ▸).

**Figure 1:**
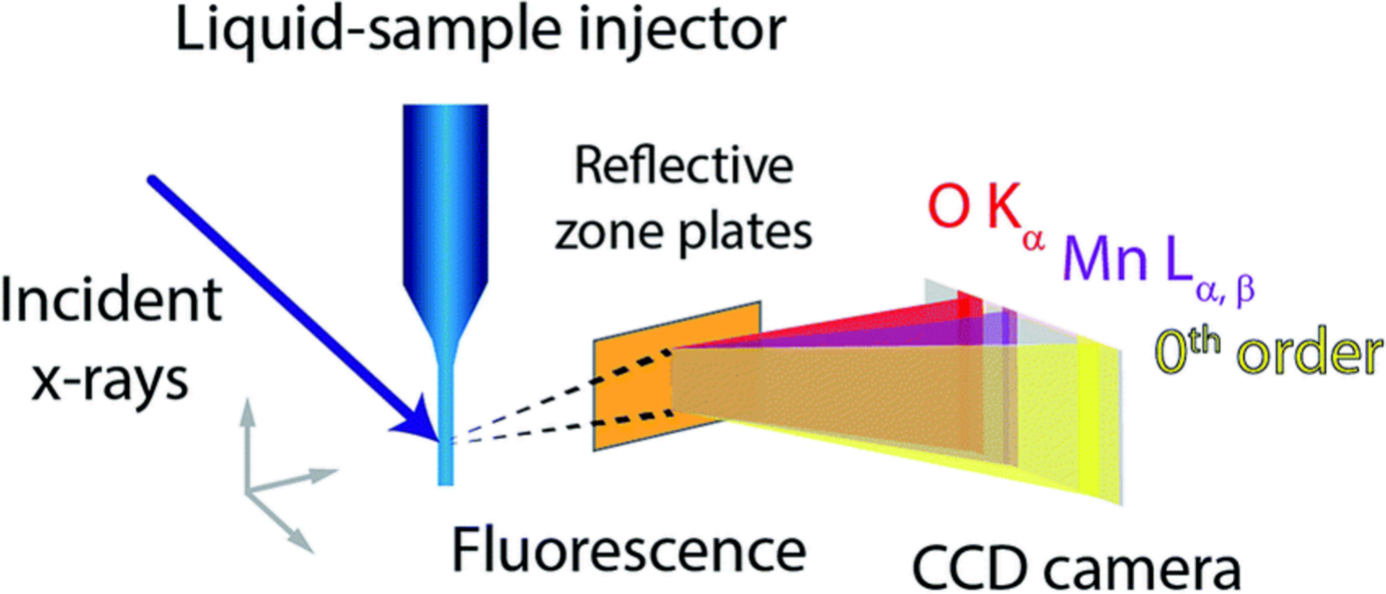
Representation of our soft X-ray fluorescence spectrometer designed for partial-fluorescence yield X-ray absorption spectroscopy. The setup incorporates a reflection zone plate (RZP) as the diffracting optical element and a detector to capture the metal *L*-edges as shown. The RZP and the CCD detector are attached and their positions can be adjusted relative to the sample via step motors. Figure taken from Kubin *et al.* (2018 ▸).

The spectrometer employs a single reflection zone plate (RZP) (Braig *et al.*, 2014 ▸) as the sole optical element to differentiate the faint metal fluorescence signal from the simultaneous oxygen fluorescence. This capability allows us to detect, for example, the manganese fluorescence signal, even in cases where the manganese-to-oxygen ratio is heavily skewed towards the latter (Kubin *et al.*, 2017 ▸). Currently, we are utilizing our spectrometer to assess various RZP designs, both on planar and spherical substrates (single and multiple zone plates per substrate) with different concentrations of manganese to validate the design decisions we have made. Fig. 1 ▸ shows a diagram of our spectrometer, showing the component parts.

To conduct these experiments, we connect our mobile spectrometer to an open port beamline at BESSY II and align the RZP and camera with respect to the manganese sample. This alignment process is necessary when the experiment begins and whenever either the sample or the RZP is changed. In metalloproteins, the oxygen concentration is of the order of 55 *M* (mol l^−1^) while the transition metal often has a concentration of or below 1 m*M* (mmol l^−1^). Given this difference of four to five orders of magnitude, precise alignment of the spectrometer is particularly important when targeting the fluorescence of the transition metal. Additionally, X-ray free-electron laser (XFEL) based spectroscopy requires particularly fast alignment, given the scarcity of beam time, and consequently we expect our method to be well suited for XFEL-based studies.

Achieving precise alignment of the reflection zone plate with respect to the sample and detector typically demands approximately one hour of skilled operation. The alignment process involves a meticulous nine-point procedure. Initially, a ‘grid-search’ method is applied to locate two reference markers. Subsequently, an iterative approach is employed, aligning the *x*-axis with the markers while adjusting the *y*- and *z*-axes. In most instances, this procedure effectively aligns the spectrometer. Detailed documentation of this entire process is available in Appendix *A*[App appa]. In contrast, our machine learning approach can achieve an alignment with an accuracy of roughly 0.3 mm in less than 20 s plus the time required to acquire reference measurements. This position is then sufficient to be either further refined manually or via continued optimization in order to achieve a precise alignment. As a result, it can be deployed to conserve valuable beam time.

The machine learning method we have developed operates by employing an optimiser with the objective of determining seven parameters: the absolute values of *x*, *y* and *z*, corresponding to the optimal coordinates of the RZP relative to the sample, camera offsets in the *xy*-coordinate plane of the detector, the ratio of manganese to oxygen and an intensity scaling between simulation and experiment. The greatest hurdle that our method aims to overcome is the large disparity between simulation and real-world experiment data, demonstrating that machine learning algorithms derived entirely by simulation are applicable in the real world. Similar strategies have been utilized in determining optimal beam positions at beamlines (Rebuffi *et al.*, 2023 ▸; Zhang *et al.*, 2023 ▸; Morris *et al.*, 2022 ▸). Typically, these methods involve running the optimiser on real-time measurements acquired during beamline operation, iteratively refining the beam position until the optimiser converges to an optimum. Further work in this regard has been undertaken demonstrating the use of Bayesian optimization for the alignment of beamlines and demonstrated using a digital twin (Morris *et al.*, 2023 ▸).

In contrast, our approach involves training a surrogate model using simulated data and subsequently determining the offset between simulation and reality to derive the best possible alignment. This method offers several possible advantages. Firstly, it can be developed and refined offline using simulation data, eliminating the need to acquire beam time for development. Secondly, the trained surrogate model can be applied beyond alignment, for instance, optimizing design parameters. Thirdly, the application at the beamline can be tuned to the accuracy level required for the given experiment. For example, if more accuracy is required, then the user has the option to feed more experiment images into the algorithm or to run the optimization process for longer, affording the optimiser the ability to further refine the alignment. This innovative approach enhances efficiency and flexibility in experimental planning and execution.

## 2. Method

The automated alignment method we have developed is a simple four step process (the first two steps are performed in advance in an ‘offline’ capacity):

(i) Simulate the setup using our in-house-developed *RAYX* software (see Subsection 2.1 for details).

(ii) Train the neural network using the simulated dataset, learning a mapping between the simulated *x*,*y*,*z* coordinates, camera offset values, and a ratio of manganese to oxygen and the resultant image.

(iii) Using the spectrometer, record *n* measurements covering the search space (approximately 10 to 25 is sufficient).

(iv) Run an optimiser with the goal to minimize the average difference between the recorded measurements and the prediction of the neural network, whilst optimizing the required offsets *X*-off, *Y*-off and *Z*-off, which are the target offset values, defining the optimal position for alignment as well as four further parameters (camera offsets in *x* and *y*, a ratio of manganese to oxygen and an overall intensity value).

### 2.1. Simulation

At BESSY II, we are actively developing a new open-source iteration of our existing ray tracing software, *RAY-UI* (Baumgärtel *et al.*, 2019 ▸). This new software, named *RAYX*, is crafted to harness the full capabilities of modern GPU technology while adhering to contemporary software development design patterns, thereby enhancing maintainability. *RAYX* is driven by two primary objectives: (i) to supersede *RAY-UI* for optical element and beamline design, and (ii) to facilitate the efficient generation of extensive datasets for training neural networks as surrogate models. *RAYX* is open source and available on GitHub (Maier *et al.*, 2024 ▸). The code can be built from source or installed via a release version for Linux or Windows.

Presently, *RAYX* provides a command-line interface that enables users to load simulation parameters, subsequently executing multiple instances of the simulation in parallel using either GPU or CPU computing for ray tracing. While the command-line functionality is available, ongoing efforts are directed towards the continuous development of a graphical design interface. During this transitional phase, *RAY-UI* is utilized to generate the initial XML files which define the parameters, ensuring a seamless integration between the two software tools. As the software develops, alternative input methods and formats will be integrated.

To train the neural network, we conducted one million simulations, systematically varying the *x*, *y* and *z* positions of the RZP and the detector within specific intervals (in millimetres): *x* [−5.0, 5.0], *y* [−5.0, 5.0], *z* [−5.0, 5.0], the *xy*-coordinate plane of the detector and the ratio of manganese to oxygen. These intervals align with the real-world search space, specifically representing the mechanical limits of the motors attached to the spectrometer. Notably, the *z*-axis does actually have a broader range, serving as the ‘zoom’ axis toward the detector. Movement along the *z*-axis imparts significantly less visual variation to the diffracted image compared with movements along the *x*- and *y*-axes and therefore it was considered preferable to limit this range to the equivalent for the *x*- and *y*-axes.

Regarding the dataset, initial tests were performed using 200000 simulations; however, the decision to generate a dataset of one million simulations was ultimately taken due to the authors’ prior experience in training similar deep neural networks and the goal of creating a more robust network capable of a greater level of accuracy during inference. This exact number is ultimately rather arbitrary, but in most cases more data are of benefit to help the network generalize (Mahajan *et al.*, 2018 ▸). Affording the model access to more diverse and representative data means it is less likely to overfit, because it has a larger and more varied set of examples to learn from. This is particularly true if the additional data contains variations and complexities that are reflective of the real-world scenarios the model will encounter, which given the intricacies of the signal generated by our RZP lead us to requiring more data. Given that this work was conducted away from the beamline and simulated positions were randomized, the option to generate even more additional simulations existed but was considered unnecessary. The chosen dataset size of one million was deemed sufficient to achieve the desired level of network robustness for accurate inference.

### 2.2. Augmentation

To minimize the inherent disparity between the network predictions and the actual experimental recordings, we employed data augmentation techniques. Prior to training the network with simulated data, we introduced *x* and *y* camera offsets, shifting the position of the image in the 2D-plane of the detector. Additionally, we varied the ratio of manganese to oxygen in the simulation by scaling the resultant intensities. These artificial augmentations were essential to ensure the method’s applicability independent of factors that might fluctuate from beam time to beam time.

The inclusion of camera offsets addresses potential variations in the exact camera position relative to the RZP, which can differ based on the spectrometer setup. Therefore, the network, during training, must also receive detector positions in *x* and *y* in order to successfully generate an image which conforms with the experiment. Similarly, the absolute intensities of the measured manganese and oxygen signals are deemed critical and should be correctly predicted. Although the primary focus is on capturing the form and position of the signal, optimizing these extra parameters enhances the robustness of the network across varying experimental conditions.

### 2.3. Neural network

The surrogate model is implemented as a standard multi-layer perceptron, and its architecture was intentionally kept as compact as possible while still achieving satisfactory results. This design choice prioritizes fast inference, a crucial factor for optimizing efficiency in the overall process. Training and validation were conducted using purely simulated data.

To enhance the training process, a custom learning rate scheduler was employed, data normalization was applied to scale values between 0 and 1, and the dimensionality of simulated images was reduced from (256, 1024) to (64, 256). The Adam optimiser was utilized for network optimization, and mean squared error was employed to calculate both training and validation losses. The network was trained using the widely adopted PyTorch deep learning library (Paszke *et al.*, 2019 ▸). The specific architecture of the network can be found in Appendix *B*[App appb].

The trained network serves as a surrogate model for the simulation, offering the advantage of rapid predictions, taking only milliseconds instead of seconds. This efficiency makes the optimization process more feasible in comparison with simply using the simulation software within the optimization loop directly. Without using a neural network as a surrogate model, the optimization process would take multiple orders of magnitude longer, with every optimization step requiring seconds to simulate a result, compared with milliseconds for the inference of the neural network. The network takes as input 3D coordinates (*x*, *y*, *z*), camera offset values (*x* and *y*), and a ratio of manganese to oxygen. The output of the network is a 1D vector that can be reshaped to create a 2D histogram with dimensions (64, 256).

### 2.4. Image acquisition

The detector utilized in this experiment generates 2D histograms with dimensions (256, 1024). After thorough experimentation, we determined that at least ten images are necessary for the optimization process to converge successfully and with around 25 we yield decent results. These images are captured at varying motor positions and were chosen across a grid covering the entire search space and with an exposure time of 10 s. Attempts with fewer than ten measurements resulted in the failure of all tested optimisers to achieve the desired outcome. Conversely, using more than 25 measurements did not notably enhance the accuracy of the result.

The process of obtaining these images takes approximately five minutes, encompassing the time required for the motors to reposition the RZP and the acquisition time of the detector. It is important to note that this duration may vary based on the specific coordinates provided, influencing the movement requirements of the RZP and the overall acquisition time.

### 2.5. Optimization

In order to ascertain the optimal alignment, coordinate optimization is required. Given *n* images acquired at the experiment at varying positions and a surrogate model trained with simulation data (NN), a loss function can be defined as follows, 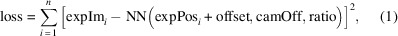
where

1. loss := difference to minimize;
2. expIm := detector image captured at experiment;
3. NN := neural network [NN(parameters) returns the predicted image from the neural network];
4. expPos := *x*,*y*,*z* position of detector image;
5. offset := *x*,*y*,*z* offset between experiment and simulation;
6. camOff := *x*,*y* detector position offset;
7. ratio := ratio of manganese to oxygen.

The loss function represents the difference between the experimental images and the network’s predictions. The primary objective of the optimiser is to minimize the disparity between the experimentally acquired measurements and the predictions generated by the neural network by determining the optimal linear offset values for the three axes, *x*, *y* and *z*, as well as the camera offsets, the ratio of manganese to oxygen and the intensity scaling factor. Once the optimization process has completed successfully, the derived offset signifies the absolute optimal alignment position of the RZP and camera relative to the sample within the spectrometer. The accuracy of the simulations in combination with the applied augmentations and consequently the quality of the trained neural network play a key role in how successfully the optimiser can achieve this. In particular, the form of the manganese and oxygen components of the signal are significant and these need to match as closely as possible between simulation and experiment for this process to succeed. If, for example, adjusting the *x*, *y* and *z* parameters in the simulation does not create an equivalent linear shift in the real spectrometer, then the offset values attained by the optimization process will not equate to the optimal alignment of the spectrometer.

**Figure 2:**
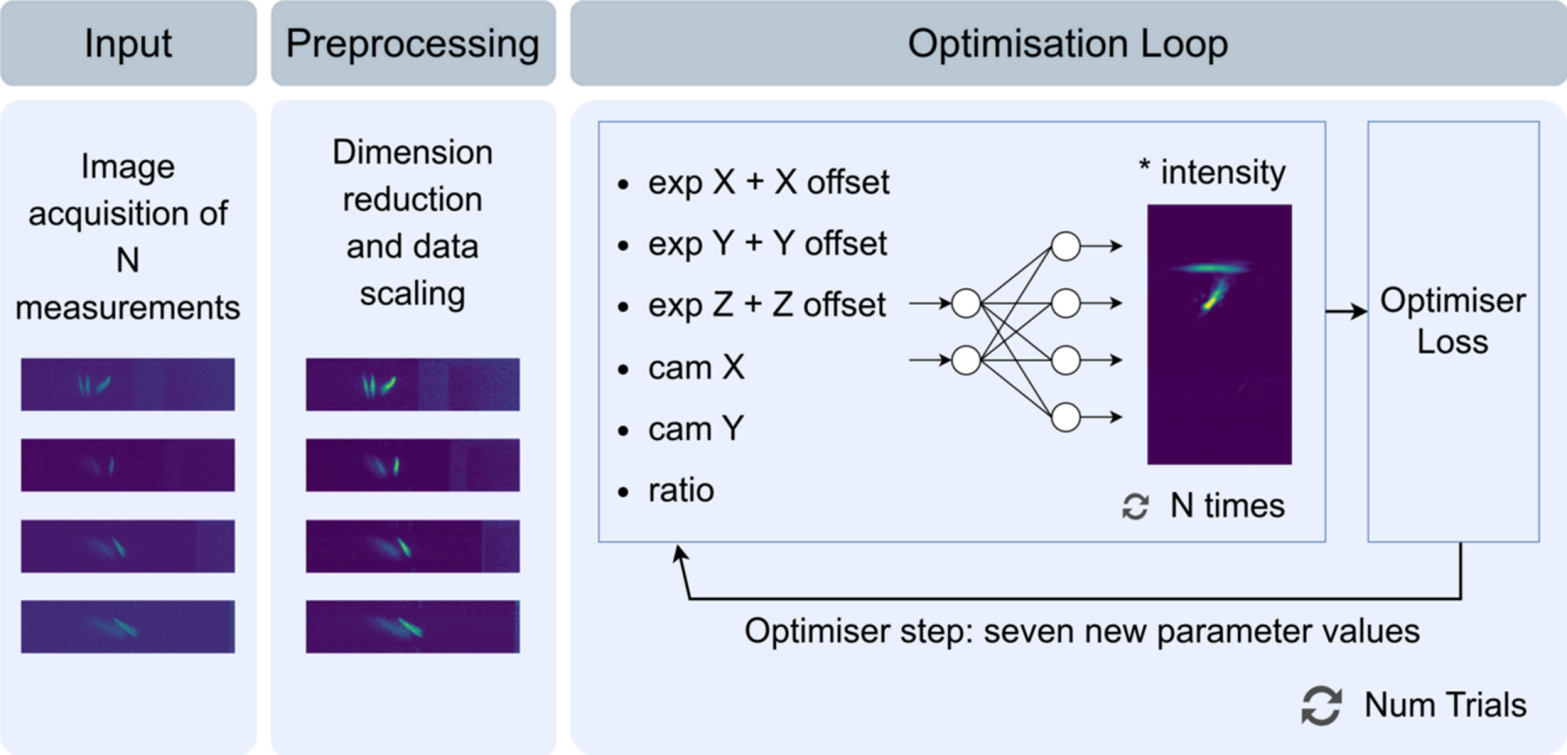
Pipeline for obtaining the optimal values for the seven parameters (the six neural network input parameters and the intensity scaling value). The two deciding factors as to how long the optimiser tries to find a global optimimum are: *N*, the number of acquired measurements; and Num Trials, a parameter set by the researcher and optimiser-dependent.

During the optimization process, the parameters camOff and ratio are also optimized as the neural network has been trained to consider variations in these factors. Additionally, a scaling factor is determined so the variance in overall intensity between the experimental images and the predictions of the network can be synchronized. Once the optimiser has successfully converged, the alignment process is considered complete, and the determined offset values represent the desired result. These values signify the optimal configuration that minimizes the difference between the actual experimental measurements and the predictions provided by the neural network. Fig. 2 ▸ shows this optimization loop in practice.

## 3. Results

The validation of our method was conducted at the UE52-SGM beamline at BESSY II (Miedema *et al.*, 2016 ▸). The spectrometer was positioned at the open port, and a small 4 mm-long wire composed of Cu86, Mn12 and Ni2 was employed as the test sample. A +1-order singular planar zone plate (produced in collaboration with Institut für Angewandte Photonik e.V.) served as the diffracting element, and a Greateyes CCD detector (GE-VAC 1024 256 BI UV1, Greateyes GmbH, Germany) captured images at a resolution of 1024 × 256, with a pixel size of 26 µm × 26 µm and a 16-bit dynamic range. Alignment of the RZP was required once the sample was positioned in the photon stream.

For data acquisition, ten sets of recordings were obtained, each comprising 25 measurements. These sets explored positions around the optimal alignment but did not include it, utilizing random absolute positions. Various optimisers were tested, and three proved to be the most effective: a Basin–Hopping optimiser (Wales & Doye, 1997 ▸), a tree-structured Parzen estimator (TPE) (Bergstra *et al.*, 2011 ▸), and simulated annealing (Kirkpatrick *et al.*, 1983 ▸). The Basin–Hopping optimiser produces the most accurate result but requires a run time of around 10 to 20 min (depending on compute hardware) compared with 20 to 30 s for the other optimisers. Our experimentation using the TPE and simulated annealing optimisers produced comparable results; however, the simulated annealing optimiser proved easier to tune and therefore was ultimately chosen.

The simulated annealing optimiser was implemented using the Optuna framework (Akiba *et al.*, 2019 ▸). To ensure the success of the optimization process, we tuned the optimiser’s hyperparameters, such as temperature, cooldown rate, neighbour range factor and number of trials. The temperature parameter is used to control the level of randomness involved in the search. A high value results in more randomness as the optimiser is more likely to accept a solution that is worse than the current best solution, whereas a low temperature value prioritizes better solutions. The cooldown rate controls how fast the temperature decreases during the optimization process, or, in other words, the rate at which the algorithm adapts its behaviour from performing a random search to focusing on refining promising solutions. Additionally, the neighbour range factor can be adjusted to control the size of the neighbourhood, the surrounding search space from any one position, where the algorithm searches for solutions. These parameters are therefore responsible for maintaining a balance between global search space exploration and local search space exploitation. After tuning, the number of trials was set at 1000, the temperature and cooldown rate at 1000 and 0.9, respectively, and the neighbour range factor to 0.1.

**Figure 3:**
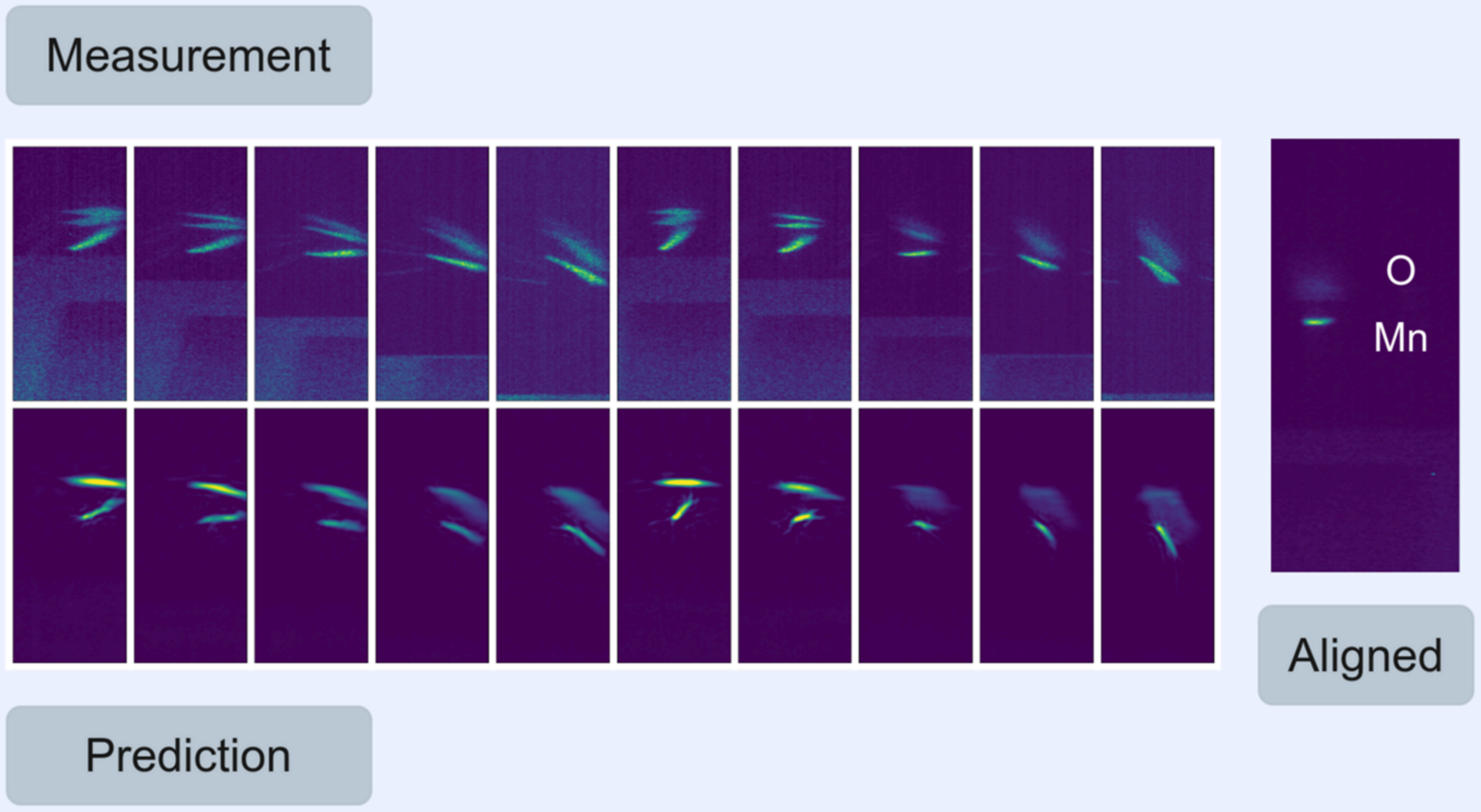
Example output of an optimization run using a simulated annealing optimiser and the Optuna framework. The loss is the average difference of the measured images in row one and the predictions of the neural network in row two. By minimizing this loss the offset between experiment and simulation is derived, which represents the position of the motors to achieve alignment. An example of this perfect alignment is shown with the manganese and oxygen components of the signal marked.

Upon convergence of the optimiser and obtaining the correct *x*, *y* and *z* offset values as well as the camera offsets, ratio and intensity values, the alignment problem was considered solved. Visualizing this outcome involved plotting the neural network predictions with the correct offset applied alongside the experimental images, constituting the pairs used for calculating the loss. An example of this visualization is presented in Fig. 3 ▸. The network accurately predicts the form and position of the manganese and oxygen components with remarkable precision. The fidelity of the images generated by the network do not match the experimental images due to the fact that the network is trained using simulation data. Additional factors such as normalization, dimensionality reduction and the effective disregarding of irrelevant data such as noise and zero-order light also contribute. Nonetheless, the prediction is robust enough to achieve the desired level of accuracy and demonstrates that the correct offsets for the *x*-, *y*- and *z*-axes have been established and these values can be used to align the spectrometer. This aligned position is also shown in Fig. 3 ▸ and results from moving the motors of the spectrometer to the acquired offset position.

**Figure 4:**
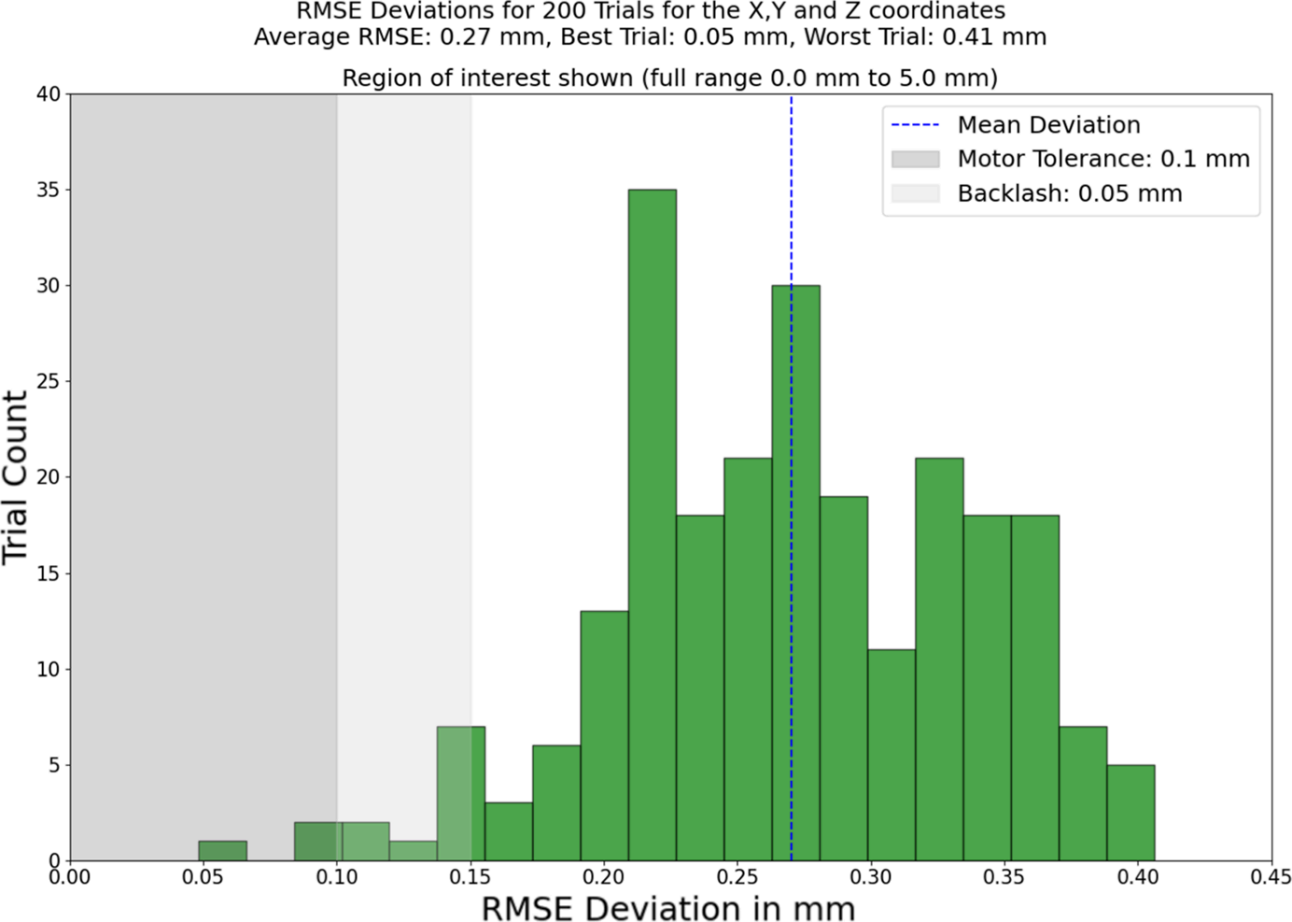
Result of performing 200 trials and calculating the RMSE of the resulting coordinates and the target for the optimal position. The *x*-axis is truncated to show just the region of interest, because all deviations are less than 0.45 mm, despite a maximum possible deviation of 5.0 mm. The step motors on the spectrometer have a tolerance of 0.1 mm and a backlash effect of roughly 0.05 mm which are shown. The average deviation is 0.27 mm with regards to an interval of −5.0 mm to 5.0 mm, with an average run-time of 19.0 s.

To account for the inherent randomness in optimization, we assessed the robustness of our approach through 200 trials, all conducted using the same dataset (a single set of 25 recordings), but with a different seed. This results in each trial starting with different values for the seven parameters that are to be optimized. Subsequently, we computed the root mean square error (RMSE) for the predicted optimal alignment positions across the *x*-, *y*- and *z*-axes, along with the target, for each trial. The average deviation across these trials was 0.27 mm within an optimization range of [−5.0, 5.0]. All trials are within 0.41 mm of the target result, despite a maximum possible deviation of 5.0 mm. The step motors on our spectrometer have a tolerance of 0.1 mm and a backlash effect of 0.05 mm (representing an inaccuracy in the recorded position when moving in one direction). Therefore, achieving a result more accurate than 0.15 mm is deemed unnecessary, and a result within several tenths of a millimetre is considered sufficient for a rough alignment of the spectrometer. The average run-time for these trials was 19.0 s. These detailed results are presented in Fig. 4 ▸, which indicates the level of accuracy achieved despite the large range of possible staring values.

**Figure 5:**
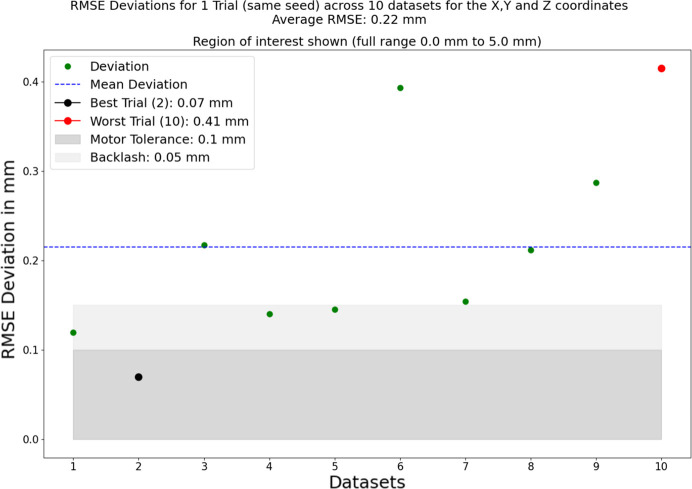
In order to test the robustness of the method, ten different datasets were taken during the beam time, each consisting of 25 measurements exploring the search space. Shown here are the RMSE of the resulting coordinates and the target for the optimal position. The *y*-axis is truncated to show just the region of interest, because all deviations are less than 0.45 mm, despite a maximum possible deviation of 5.0 mm. The average deviation is 0.22 mm, which is in line with the average deviation of one dataset across 200 trials of 0.27 mm, shown in Fig. 4 ▸.

To address variance in the experimental images, we obtained ten distinct datasets, each serving as input for the optimization process. Despite all ten datasets being captured under the same setup and during the same beam time, variations in the positions of measurements resulted in entirely unique datasets with no overlap. Mirroring our previous approach, we computed the RMSE for the predicted optimal alignment positions across the *x*-, *y*- and *z*-axes, in addition to the target, for each dataset. The average RMSE across these datasets was 0.22 mm, consistent with the results from the preceding 200 trials. This underscores that any set of input experiment images should yield successful results, provided they adequately cover the search space. The findings from this validation process are presented in Fig. 5 ▸, which indicates the level of accuracy achieved. Similarly to Fig. 4 ▸, a region of interest is shown, this time with the *y*-axis truncated.

## 4. Discussion

In the context of our mobile spectrometer, the solution we are presenting enables the automated alignment of the RZP and detector. Compared with our previous procedure, the reduction in alignment time from approximately one hour to five minutes (image acquisition time plus optimization process) represents a significant improvement and provides us with the advantage of more usable beam time.

It is important to note that the performance of the optimiser heavily relies on the randomly selected starting position, leading to variations in the optimization process. As illustrated in Fig. 4 ▸, the optimal outcome exhibited an impressive accuracy of 48.1 µm, while the least favourable result was 406.0 µm. In instances where the result falls on the latter end of the spectrum, additional manual fine-tuning may be necessary. To address this challenge, we explored the feasibility of utilizing the rapidly obtained outcome from the simulated annealing optimiser (completed in less than 20 s) as the initial position for a more systematic and exploratory Basin–Hopping optimiser. This approach proved successful, with the Basin–Hopping optimiser consistently converging to a result with an accuracy of approximately 0.1 mm. However, it is worth noting that this enhanced accuracy came at the expense of an extended run-time of around ten minutes, counteracting the initial objective of efficiency. Consequently, further testing and optimization are required in this regard.

This work demonstrates the feasibility of creating a surrogate model for complex photon science experiments using only simulation data and successfully applying it to a real-world experiment. The model is trained to take the *x*, *y* and *z* coordinates, the camera offsets and the ratio of manganese to oxygen as input and return the corresponding detector image, requiring an optimiser to determine the offset between simulation and experiment. An alternative solution could involve training an inverse network (image to coordinates). With such a model, offsets could be calculated using a simple linear fit, employing experiment detector images as input. However, this approach poses challenges as the network needs to process unseen experiment data despite being trained solely with simulation data. We are currently exploring data augmentation and domain adaptation methods in this regard.

Our objective at the Helmholtz Zentrum Berlin, concerning machine learning, is not to create custom solutions applicable only to one instrument. Instead, we aim to develop methods that can be generalized across various aspects of photon science. In this respect, this work serves as a prototype for data-driven alignment procedures applicable not only to spectrometers but also to entire beamlines and detectors in general.

## Appendix A Manual alignment procedure

(1) At the outset, shed light on the CCD through a coarse alignment of the spectrometer’s *z*-axis.

(2) Precisely centre the light on the CCD by adjusting the spectrometer’s *y*-axis. Utilize coarse tuning in 1 mm steps, followed by fine-tuning with 0.2 mm increments.

(3) Optimize the *x*-position of the Mn wire while monitoring the counts on the CCD image.

(4) Move the piezo crawler to the ‘DOUBLE HOLE’ position (10.5 mm) – a double aperture. Adjust the spectrometer’s *z*-axis and *y*-axis to locate an image of the holes.

(5) Centre the image at the half height of the CCD using the spectrometer’s *y*/*z* axes.

(6) Verification step. Shift the spectrometer’s *z*-axis by +1.7 mm and then by −1.7 mm. If, at d*Z* = −1.7 mm, another faint image of two holes (reflection) can be observed, this confirms that the initial ‘true’ transmitted image of two holes existed. If no reflection image is seen, revert to the previous *z*-axis position and check if, at d*Z* = +1.7 mm, a brighter image of two holes can be seen. If so, the earlier image was a reflection, and you are now in the ‘true’ two holes image position. Stay at this position and proceed to Step 7.

(7) Set the piezo crawler to FILTER. Starting from the position of the ‘true’ two holes, adjust the spectrometer by d*Z* = −3.6 mm. You may now be near the final alignment.

(8) Fine-tune the alignment of the spectrometer’s *y*-axis. Straighten the stripe structure by moving the spectrometer’s*y*-axis, adjusting by d*Y* = −0.3 mm.

(9) Fine-tune the spectrometer’s *z*-axis: decrease the Mn–O separation (move the image down) by increasing *Z*, and increase the Mn–O separation (move the image up) by decreasing *Z*. Use a step size of d*Z* = 0.2 mm, and for the final fine-tuning set d*Z* = 0.1 mm.

## Appendix B Network architecture

(i) Learning rate: 1 × 10^−3^ (scheduler reduces rate by a factor of 0.5 every 100 epochs).

(ii) Adam optimiser.

(iii) MSELoss with reduction = ‘sum’.

(iv) Batch size = 64.[Chem scheme1]
