# Automated spectrometer alignment via machine learning. Corrigendum

**DOI:** 10.1107/S1600577525009622

**Published:** 2026-01-01

**Authors:** Peter Feuer-Forson, Gregor Hartmann, Rolf Mitzner, Peter Baumgärtel, Christian Weniger, Marcus Agåker, David Meier, Phillipe Wernet, Jens Viefhaus

**Affiliations:** a Helmholtz-Zentrum Berlin für Materialien und Energie GmbH Albert-Einstein-Strasse 15 12489Berlin Germany; b Uppsala Universitet 751 05Uppsala Sweden; c MAX IV Laboratory Lund University PO Box 118 SE-22100Lund Sweden; European XFEL, Germany

**Keywords:** machine learning, X-ray diffraction, instrumentation, reflection zone plate

## Abstract

Corrigendum to the article by Feuer-Forson *et al.* [(2024). *J. Synchrotron Rad.***31**, 698–705].

In the original paper (Feuer-Forson *et al.*, 2024 ▸) an optimization range of −5.0 mm to 5.0 mm was given. This was in error and the actual range is −2.0 mm to 2.0 mm. This error has been corrected and the optimization method has been revised to account for this. The method optimizes seven parameters for the alignment of the spectrometer, the *X*, *Y* and *Z* offsets, the camera position in *X* and *Y*, the ratio of Mn to O, and the overall intensity. Whilst running further experimentation, it has been recognized that the method, which compares experimental and simulated images, cannot ascertain the *Z* offset. The *Z*-axis is the ‘zoom’ axis towards the camera, and this has very little impact on the resulting signal. Consequently, the updated evaluation figures present the results for only the *X* and *Y* offsets.

The updated evaluation results are shown in Figs. 4 ▸ and 5 ▸. The optimization method has been improved to include a refinement step, and the total number of trials is 2000. The updated method requires more compute resources, using two GPUs in parallel to perform inference, and results in an average run-time of 30 s. However, the updated optimization now functions with 10 samples, as opposed to the previous 25, which reduces data acquisition time and consequently overall application time. The evaluation of 200 seeds on one dataset, shown in Fig. 4 ▸, demonstrates that the method is still robust to varying seeds. Fig. 5 ▸ shows the results of evaluating 10 different datasets, and with a median RMSE of 0.18 mm the method works with differing sample images, although one outlier dataset performed worse than the other nine.

## Figures and Tables

**Figure 4:**
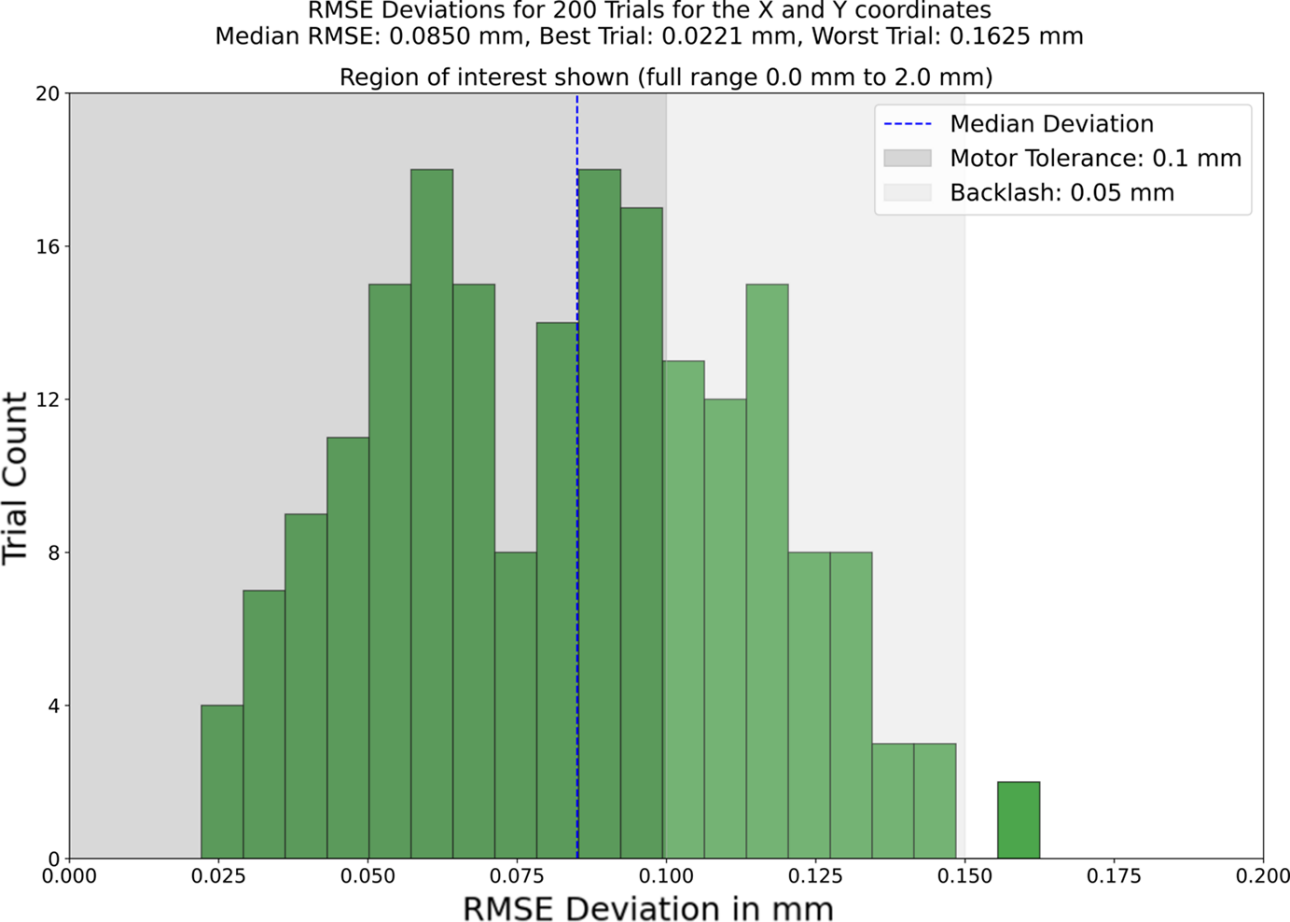
Result of performing 200 trials and calculating the RMSE of the resulting coordinates and the target for the optimal position. The *x*-axis is truncated to show just the region of interest. The step motors on the spectrometer have a tolerance of 0.1 mm and a backlash effect of roughly 0.05 mm which are shown. The median deviation is 0.085 mm with regard to an interval of −2.0 mm to 2.0 mm, with an average run-time of 30 s.

**Figure 5:**
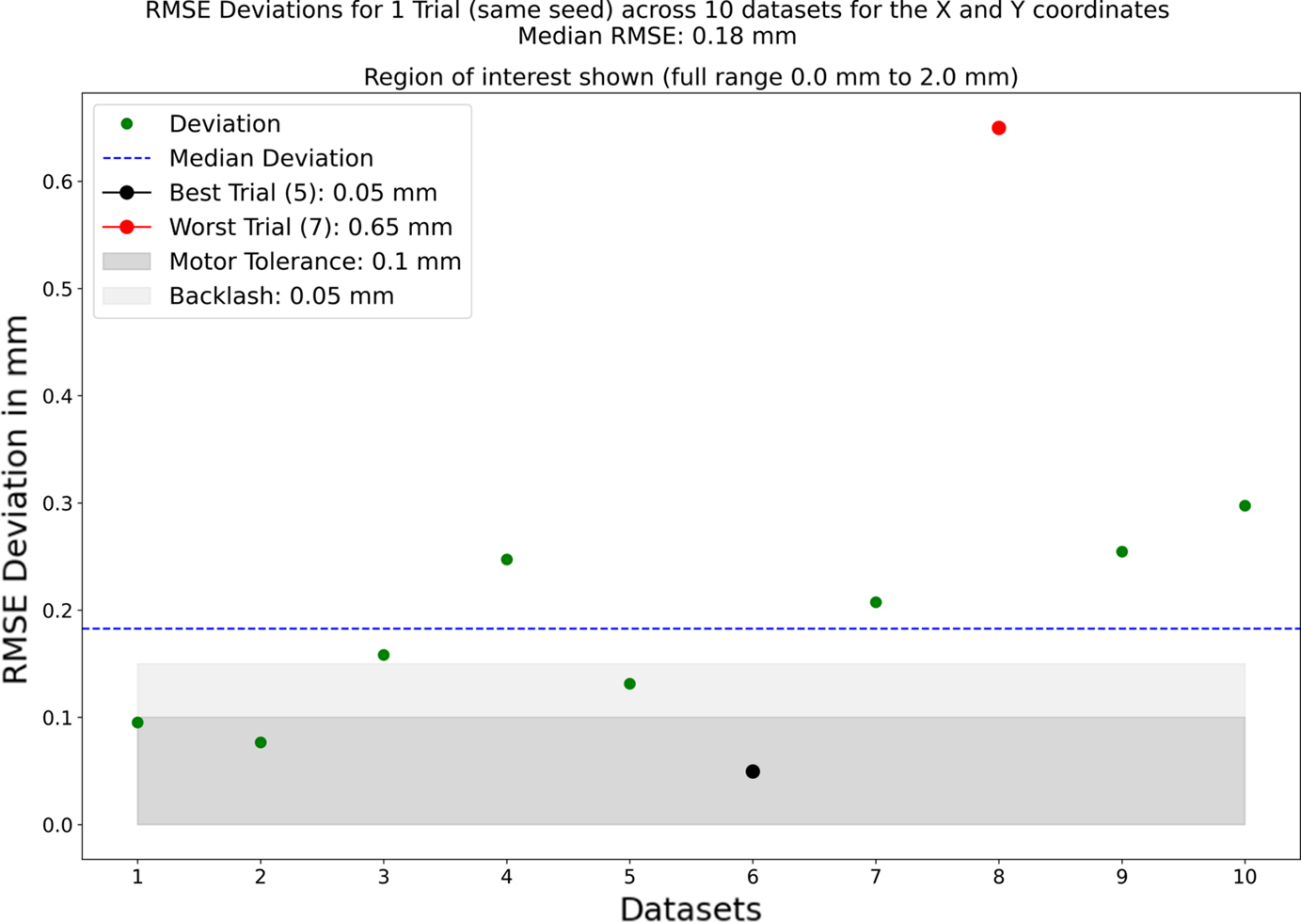
In order to test the robustness of the method 10 different datasets were taken during the beam time, each consisting of samples exploring the search space. Shown here are the RMSE of the resulting coordinates for the *X* and *Y* offsets and the target for the optimal position. The *y*-axis is truncated to show just the region of interest. The median RMSE is 0.18 mm, which is in line with the margin of error of 0.15 mm for the spectrometers motors.
